# Pharmacodynamics and pharmacokinetics of ciprofloxacin during sustained low-efficiency dialysis

**DOI:** 10.1186/cc13592

**Published:** 2014-03-17

**Authors:** K Olsen, T Plumb, N Reardon, K Bogard, A Branch-Woods, G Peitz

**Affiliations:** 1 University of Nebraska Medical Center, Omaha, NE, USA

## Introduction

Little information is available regarding ciprofloxacin pharmacokinetics and pharmacodynamics in sepsis patients receiving sustained low-efficiency dialysis (SLED). This study determined the pharmacokinetics and simulated pharmacodynamics of ciprofloxacin in ICU patients during SLED.

## Methods

This study was a prospective evaluation of ciprofloxacin pharmacokinetics in patients with sepsis and >18 years of age, urine output <200 ml/day and receiving SLED for at least 8 hours. Following informed consent, plasma samples were collected at baseline and 1, 2, 4, and 8 hours after a ciprofloxacin 400 mg dose i.v. during SLED and post-SLED therapy at the same times. Dialysate samples were collected at 4-hour intervals during SLED. Pharmacokinetic parameters were determined using WinNonlin and compared between the two periods. Simulated pharmacodynamic parameters were determined for *Pseudomonas aeruginosa *using MIC = 2.

## Results

A total of seven patients (four male, three female, age 56.9 ± 7.6, APACHE II 26.8 ± 2.4) were enrolled. Ciprofloxacin was cleared relatively rapidly with a half-life of 6.9 hours and a Ke of 0.108/hour during SLED compared with 11.9 hours and 0.057/hour post-SLED (*P *< 0.05). Simulated pharmacodynamics demonstrated inadequate coverage for *P. aeruginosa *during sLeD with Cmax/MIC ratio 5.7 ± 1.2 and AUC/MIC 77.5 ± 22.3.

## Conclusion

Ciprofloxacin is rapidly cleared during SLED similar to clearance during normal renal function, which may result in adequate pharmacodynamic coverage for some pathogens.

